# Correlation between stereoradiography and 3D topographic measurements

**DOI:** 10.1186/1748-7161-8-S2-O22

**Published:** 2013-09-18

**Authors:** XueCheng Liu, John Thometz, Carlos Marquez-Barrientos, Channing Tassone

**Affiliations:** 1 Dept. of Orthopaedic Surgery, Medical College of WI, Milwaukee, WI, USA

## Background

The EOS is a low-dose digital stereoradiography system that can produce highly reproducible spine measurements. The Milwaukee Topographic System (MTS) has reliably detected the 3-dimensional (3D) back surface contour changes. The correlation between Cobb angles and the MTS analog to Cobb angles in both the sagittal and coronal planes have been studied, but data are limited for comparisons of axial vertebral rotation measures derived from the MTS and X ray system.

## Purpose

The goal of this study was to evaluate the correlations between EOS-derived axial rotation and MTS-registered axial surface rotation at each vertebral level.

## Methods

A case control study design was performed, and four subjects with adolescent idiopathic scoliosis (ages 10 to 17) had measurements taken using both the MTS and EOS system. The MTS used a handheld laser scanner to gather the subjects' back geometry while standing. Markers were placed on the subjects' spinal processes so they could be identified in the scans and a custom software package was used to calculate 3D back measures. The EOS system took bi-planar images of the subjects’ torsos while standing and then calculated spine measurements by semi-automatically making a 3D reconstruction of the spine. The vertebral axial rotations from the MTS and EOS system were compared for the thoracic and lumbar vertebrae. The Pearson correlation coefficient was calculated for all the vertebrae.

## Results

Only one vertebral level measurement, the third lumbar vertebrae (r=0.95, p<0.049), had a significant correlation, although the eighth (r =0.94, p<0.06) and ninth (r=0.92, p<0.08) thoracic vertebrae had high correlations that were close to significance.

## Conclusions and discussion

In this limited study, the MTS predicts for the transverse rotations of the spinal segments, especially in the thoracolumbar and proximal lumbar region. Our current experience may indicate that the larger the vertebral body, the better the 3D reconstructive process using EOS.

